# Camptocormia, a Rare Complication of Hemorrhagic Stroke: A Case Report

**DOI:** 10.7759/cureus.32450

**Published:** 2022-12-12

**Authors:** Kuan Geok Ng, Qiao Qi Teo

**Affiliations:** 1 Rehabilitation Medicine/General Medicine, Sengkang General Hospital, Singapore, SGP

**Keywords:** rehabilitation, madopar, treatment, hemorrhagic stroke, camptocormia

## Abstract

Camptocormia (bent spine syndrome) is a rare complication after hemorrhagic stroke. It is a disabling, acquired postural abnormality that has a significant impact on a patient's physical function and quality of life and is more often seen in patients with Parkinson’s disease. Treatment strategies pertaining to this condition can be broadly divided into invasive which involved deep brain stimulation and spinal fixation operation versus non-invasive approaches like physiotherapy, orthosis and drugs. However, most of the treatments described in the past are mainly for Parkinson’s patients with camptocormia, and none are for camptocormia from hemorrhagic stroke. We report a rare case of camptocormia in a posthemorrhagic stroke patient whose rehabilitation progress was greatly impeded by this axial postural deformity and who responded well to Madopar treatment with an improvement in total camptocormia angle from 90 degrees to about 30 degrees, which translated to an improvement in physical function and reduction in care burden.

## Introduction

Camptocormia (bent spine syndrome) is a major, disabling, non-fluctuating acquired postural abnormality characterised by involuntarily forward flexion of the thoracolumbar spine which typically appears during walking or standing and completely disappears in a supine position [1]. Camptocormia was first documented in the 17th century by the Spanish painter Francisco de Zurbaran. ‘Functionally bent spine’ was first described by Brodie in 1818 [2]. The term “camptocormia” was first coined in 1915 by the French neurologist Rosanoff-Saloff [3] who described the abnormality in World War 1 soldiers traumatised by shell shock, combining the Greek words kamptos, which means “curved,” and kormos, which means “trunk.” There is no previous report on haemorrhagic stroke and camptocormia. Hence we would like to report a rare case of a posthaemorrhagic stroke patient with camptocormia that had greatly impeded his rehabilitation progress with significant improvement in posture after starting on Madopar.

## Case presentation

**Figure 1:**
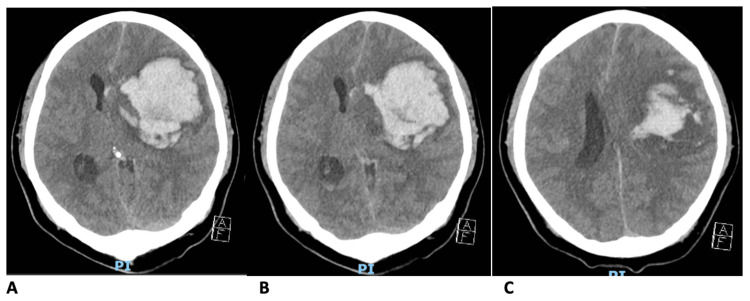
Patient’s CT brain on admission. A-C: Urgent CT brain showed large left basal ganglia hematoma with intraventricular extension, mass effect causing effacement of the left lateral ventricle and midline shift.

A Chinese gentleman in his 30s with no known medical issues at presentation was found unconscious on the floor in the morning at home. On arrival to hospital, his Glasgow Coma Scale (GCS) was E1V1M3, right pupil reactive at 2mm, left pupil was fixed and dilated at 3mm, and he was intubated at emergency department. Urgent computed tomography (CT) brain showed large left basal ganglia hematoma with intraventricular extension, mass effect causing effacement of the left lateral ventricle, midline shift and trans-tentorial herniation (Figure 1). CT angiography showed no overt aneurysm, no arterio-venous malformation and no angiographic spot sign. 

He underwent urgent left craniotomy and evacuation of left basal ganglia hematoma with insertion of intracerebral pressure wire. He was subsequently admitted to surgical intensive care unit (SICU) and had open tracheostomy due to persistent low GCS, eventually able to wean off ventilator and was transferred to general ward after 10 days of stay in both SICU and high dependency unit. He was also diagnosed with hypertension and hyperlipidemia for which he was started on treatment.

He was in an unresponsive wakefulness state with an initial JFK Coma Recovery Scale (JFK-CRS) of 3 upon transfer to the general ward. In view of his low JFK-CRS score, he was initially started on rehabilitation with regular range of motion exercises for all four limbs, regular sitting out of bed daily, multimodal sensory stimulation and verticalization with tilt table. About a month later, he showed improvement in his cognition with improvement in participation during therapy sessions, he was then started on body weight supported ambulation training, transfer practice, sitting balance training, and cognitive training as tolerated. At two months, he had an observable increased in his JFK-CRS score from 3 to 13 with improvement in motor and visual domains, and he was successfully decannulated. At three months, he emerged from disordered consciousness as he was observed to have functional object use and was able to wean off the nasogastric feeding tube.

**Figure 2:**
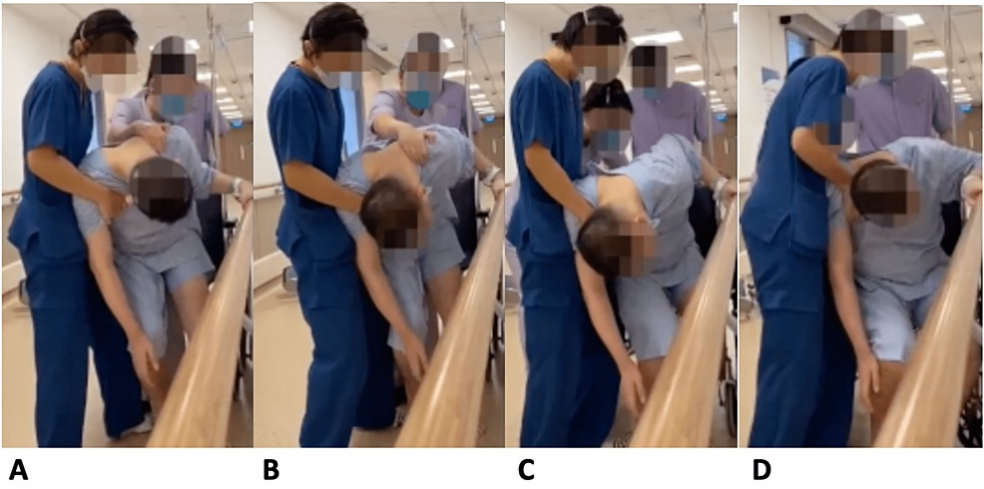
Patient's posture during ambulation before Madopar A-D: Severe forward bending posture during ambulation practice with therapists. In view of camptocormia, he required two to three people to assist with short-distance ambulation practice, and his progress was severely impeded.

We encountered a number of issues when we started him on ambulation exercises. First of all, he had spasticity over bilateral ankles. For spasticity management, he was initially put on bilateral resting ankle foot orthosis, regular functional electrical stimulation to bilateral tibialis anterior muscles with oral antispasticity medications such as baclofen and tizanidine. His oral antispasticity medications up-titration was limited by deranged liver function test and drowsiness with higher dosage, he was kept on baclofen 10mg three times a day and tizanidine 8 mg twice daily (BD). His ankle plantarflexors spasticity continue to affect his standing, he was then given total 400 units of intramuscular botulinum toxin injection over bilateral gastrocnemius and soleus muscles. Secondly, about five months after his hemorrhagic stroke, he was noted to have forward bending posture with total camptocormia angle of 90 degrees during standing despite constant verbal and tactile feedback, however, he was able to lie flat when he was in a supine position. Total camptocormia angle is the angle between the line from the lateral malleolus to the L5 spinous process and the line between the L5 spinous process and the spinous process of C7 [4]. This forward bending posture in standing greatly impeded his rehabilitation progress (Figure 2). 

**Figure 3:**
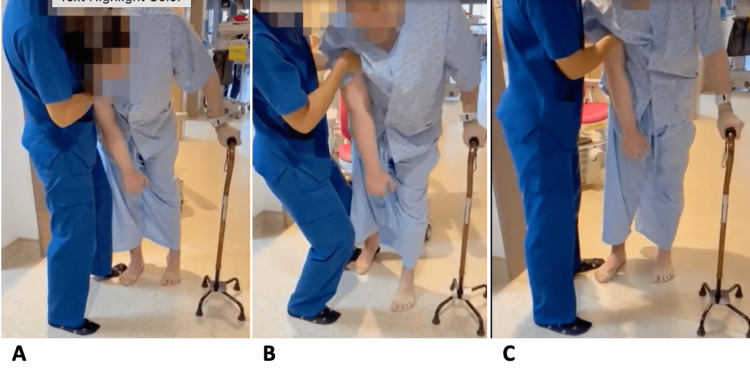
Patient's posture during ambulation after starting on Madopar A-C: The patient's forward bending posture significantly improved after starting on Madopar. He has made significant improvement in his rehabilitation, and he can now walk with one person assisting and a walking stick.

He was put on trial of thoracolumbar spinal orthosis (TLSO), however, it was not well tolerated, and he kept removing it despite repeated reminders from therapists. In view of failed oral antispasticity medications and TLSO, he was started on a trial of oral Madopar, initiated at 62.5 mg BD then slowly up-titrated over few weeks till desirable improvement in his posture observed without any significant postural drop in his blood pressure. His oral Madopar was kept at 62.5 mg four times a day as at this dose he achieved a fairly upright posture in standing with total camptocormia angle of about 30 degrees without any significant drop in postural blood pressure (Figure 3). 

An improvement in his forward bending posture in standing translated to a significant progression in his physical rehabilitation. At one-year after his hemorrhagic stroke, he was able to stand straight holding on to a metal door grill and able to ambulate with one person assisting for a short distance with a broad base quad stick. He was currently taken care of by a caregiver at home. 

## Discussion

Camptocormia, also known as bend spine syndrome, is most frequently associated with Parkinson's disease. Parkinson’s disease is a degenerative, progressive disorder that affects nerve cells in deep parts of the brain called the basal ganglia and the substantia nigra. In a single-centre epidemiological study on 275 consecutive outpatients with Parkinson’s disease, the prevalence of camptocormia was 6.9% [5]. In our patient, his haemorrhagic stroke was over basal ganglia, which is one of the common locations for parkinsonism.

Other conditions associated with camptocormia can be broadly divided into central nervous system disorder (CNS), peripheral nervous system disorder (PNS), myopathies, drugs, psychiatric disorders and others (Table 1). CNS disorders included dystonia, multisystem atrophy, Alzheimer’s disease, basal ganglia disorders, and essential tremor [6,7]. PNS disorders included amyotrophic lateral sclerosis, chronic inflammatory demyelinating polyneuropathy (CIDP), myasthenia gravis and lumbar disc herniation. Myopathies included various primary and secondary myopathies, for example myotonic dystrophy, fascioscapulohumeral muscular dystrophy, hypothyroid myopathy, inflammatory myopathy, and dermatomyositis [6,7]. Examples of drugs are olanzapine, donepezil, valproate and systemic steroid [6,7]. Psychiatric disorders include conversion disorder and psychogenic [6,7]. Other causes included trauma, arthritis, malignancies and idiopathic causes [6,7].

**Table 1:** Etiologies of camptocormia

Central nervous system (CNS)
Parkinson’s disease
Dystonia
Multisystem Atrophy
Lewy body disease
Frontotemporal dementia
Progressive supranuclear palsy
Alzheimer’s disease
Basal ganglia disorder
Essential tremor
Peripheral nervous system disorder (PNS)
Amyotrophic lateral sclerosis
Chronic inflammatory demyelinating polyneuropathy (CIDP)
Myasthenia gravis
Lumbar disc herniation
Cervical myelopathy
Myopathies
Myotonic dystrophy
Fascioscapulohumeral muscular dystrophy
Hypothyroid myopathy
Mitochondrial myopathies
Inclusion body myositis
Radiation myopathy
Inflammatory myopathies
Dermatomyositis
Drugs
Olanzapine
Donepezil
Valproate
Systemic steroid
Psychiatric disorders
Conversion disorder
Psychogenic
Others
Trauma
Arthritis
Malignancies
Idiopathic

Camptocormia associated with a CNS disorder usually is a result of focal action dystonia of the spine. The CNS structure that is affected is the striatum and its projection to the reticulospinal tract or the thalamus. This is supported by beneficial effect of deep brain stimulation on camptocormia and the reduced midbrain and pons volume in patients with Parkinson’s disease and camptocormia [8]. For PNS and myopathies, camptocormia is usually due to involvement of the antigravity muscles associated with trunk extension. This is supported by myopathic changes on paravertebral muscle electromyography (EMG), vertebral muscle MRI, and muscle biopsy [8].

**Figure 4:**
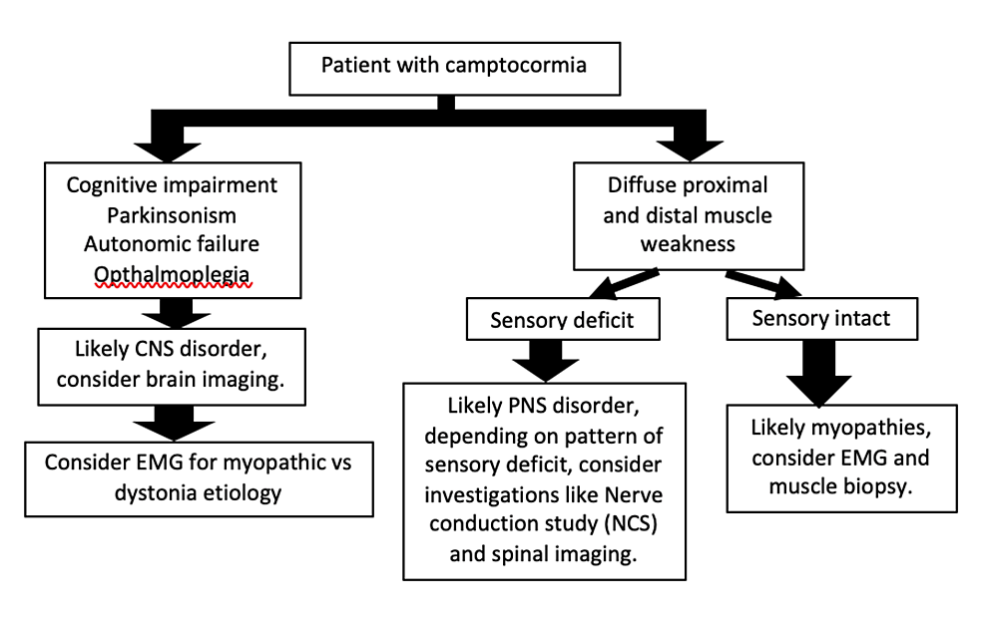
Diagnostic work up for Camptocormia CNS: central nervous system, EMG: electromyography, PNS: peripheral nervous system Figure created by Kuan Geok Ng

Since the aetiology of camptocormia is quite heterogeneous, investigations in different directions have to be carried out at the beginning of the diagnostic workup (Figure 4). Diagnosis may be established upon clinical findings, blood investigations, imaging, electromyography (EMG) or muscle biopsy. Blood investigations to look for metabolic myopathy such as blood C-reactive protein, electrolytes (calcium and phosphorus), muscle enzymes (creatine kinase, aldolase) and vitamin D [6,7]. Cerebral imaging may show basal ganglia and lenticular lesions such as atrophy, calcification, lacunas, or reduced volume of the midbrain or pons [6,7]. Depending on the underlying aetiology, EMG can be normal, neurogenic or myogenic. Muscle biopsy may be normal or may show myopathic features, inflammatory features suggesting focal inflammatory myopathy (focal myositis), or dystrophic features [6,7]. For our patient, his blood investigations were unremarkable, EMG and muscle biopsy were not done as he has known massive left basal ganglia haemorrhagic stroke that likely can account for his camptocormia given the onset of his camptocormia was after his massive haemorrhagic stroke. 

As mentioned earlier, for CNS disorder, camptocormia is usually a result of focal action dystonia of the spine. According to National Institute of Neurological Disorder and Stroke (NIH), dystonia is a disorder characterized by involuntary muscle contractions that cause slow repetitive movements or abnormal postures. There are many different forms of dystonia such as generalised dystonia, focal dystonia, multifocal dystonia, segmental dystonia and hemidystonia. They are grouped by the region of the body which they affect. Task-specific dystonia tends to occur only when undertaking a particular repetitive activity, for example musician’s dystonia, which is a term used to classify focal dystonia affecting musicians, specifically their ability to play an instrument or to perform. For camptocormia, it is likely a form of task-specific focal dystonia that happens when the patient stands or walks. 

Our patient has spastic dystonia as he has both spasticity and dystonia. Spasticity is defined by Lance in 1980 [9] as a velocity-dependent increase in tonic stretch reflexes with exaggerated tendon jerks, resulting from hyperexcitability of the stretch reflex. Spastic dystonia is the inability to relax a muscle leading to spontaneous tonic contraction. Patients with spasticity are able to keep their muscles relaxed prior to muscle stretch, however, this is not observed in patients with dystonia. In this case, he was already on oral anti-spasticity medications, unfortunately, the side effects of the medications prevent further optimization of the dose. It is important to treat camptocormia as it will affect the patient’s function. A study on camptocormia in Parkinson’s disease showed that patients with camptocormia tend to show lower scores for sleep, fatigue, attention and memory and treatment may improve their quality of life (QOL) [10]. For our patient, his physical rehabilitation progress in term of transfer and ambulation was clearly impeded by camptocormia, hence is important for us to initiate treatment.

Treatment options can be broadly classified into conservative or invasive (Table 2). Treatment of choice is the therapy of the underlying disorder and withdrawal of causative drugs if any. In cases where no disease-modifying agents are available, orthoses, physiotherapy, and analgesics are the only choice [7]. Psychotherapy includes psycho-education regarding secondary gain, suggestions to improve posture, positive reinforcement, or behavioural therapy [7]. Application of a thoracopelvic anterior distraction orthosis results in a QOL increase by 90% [11]. However, orthoses often poorly tolerate and quickly abandoned. Camptocormia associated with Parkinson’s disease, dystonia, or multiple system atrophy (MSA), administration of levodopa has been shown to be beneficial [12,13]. However, for advanced Parkinson’s disease, camptocormia is usually levodopa resistant. Steroid administration showed a beneficial effect in a single case of paraspinal muscle inflammatory myopathy [7]. Botulinum toxin injection into the rectus abdominus muscle and iliopsoas muscle may relieve camptocormia in patients with focal dystonia and Parkinson’s disease [14,15]. A systemic review suggested deep brain stimulation was an effective treatment option for Parkinson’s disease-related camptocormia [16]. In cases where conservative measures are unsuccessful, patients may benefit from posterior thoracolumbar fixation, however, major complications following spinal surgery were noted and the patient required multiple revision operations [16].

**Table 2:** Treatment of camptocormia

Non-Invasive
Psychotherapy
Physiotherapy
Orthoses
Backpack treatment
Antidystonia medication
L-Dopa
Steroids
Botulinum toxin
Invasive
Deep brain stimulation
Posterior thoracolumbar fixation
Anterior interbody fusion

For our patient, he had tried few of the non-invasive interventions. He tried thoracolumbar spine orthoses for therapy sessions, however he was unable to tolerate it, and he kept removing them despite being repeatedly reminded by therapists. He was on antidystonia medications such as baclofen and tizanidine, however there was no improvement in his standing posture. He was not a suitable candidate for psychotherapy in view of severe cognitive impairment. Botulinum toxin (BOTOX, total 400 units) was given to bilateral gastrocnemius and soleus for severe bilateral lower limbs spastic dystonia with ankle in plantarflexion. Botulinum toxin was not given to rectus abdominis and iliopsoas muscle as lack of expertise in our centre to give botulinum toxin in such locations, and he had reached the ceiling of treatment for botulinum toxin injection as maximum cumulative dose should not exceed 400 Units in a three-month interval [17]. As we had exhausted the treatment options for our patient, we decided to start him on a therapeutic trial of Madopar given his hemorrhagic stroke location was over the basal ganglia which is the same brain area that is involved in the pathogenesis of Parkinson’s disease. Previous studies have suggested potential beneficial effect of Madopar for treatment of camptocormia in Parkinson’s disease. Madopar is a combination of levodopa and the decarboxylase inhibitor benserazide (as hydrochloride) in a ratio of 4:1 [18]. It is a well-known oral treatment for Parkinson’s disease, listed as one of the oral treatment options for dystonia in the National Institute of Neurological Disorder and Stroke (NIH) website. The patient had noticeable improvement in his standing and ambulation posture after starting on Madopar, and dosage was slowly up-titrated and kept at 62.5mg four times a day in view of postural hypotension with higher dosage.

## Conclusions

Camptocormia is a very rare debilitating complication of haemorrhagic stroke, a form of task-specific focal spastic dystonia. It has a significant impact on a patient’s physical rehabilitation process, particularly in transfer and ambulation. Successful treatment can translate into improvement in physical function, reduction in care burden and potentially better quality of life for both patient and caregiver. From our observation, physiotherapy intervention with body weight-supported ambulation training and feedback coupled with pharmacological treatment (i.e. Madopar) are beneficial to patients with such presentation. Our patient achieved clinically significant improvement in total camptocormia angle from 90 degrees to about 30 degrees. This is associated with an improvement in physical function with reduction in care burden which allowed him to be discharged back to the community.
